# THE RELATIONSHIP BETWEEN DYADIC COPING AND INTIMACY OF PATIENTS WITH STROKE AND SPOUSES: A DYADIC APPROACH

**DOI:** 10.1093/geroni/igae098.3396

**Published:** 2024-12-31

**Authors:** Xiaohui Liu, Nana Liang, Xuan Du, Haihua Gao, Xiaoping Yang, Huijuan Wang, Jialin Yuan

**Affiliations:** Ningxia Medical University, Yinchuan, Ningxia, China (People’s Republic); Gansu Medical College, Pingliang, Gansu, China (People’s Republic); HeXi University, Zhangye, Gansu, China (People’s Republic); Ningxia Medical University, Yinchuan, Ningxia, China (People’s Republic); Ningxia Medical University, Yinchuan, Ningxia, China (People’s Republic); Ningxia Medical University, Yinchuan, Ningxia, China (People’s Republic); Ningxia Medical University, Yinchuan, Ningxia, China (People’s Republic)

## Abstract

Most studies have focused on coping in middle-aged and elderly stroke patients and spouses in isolation. However, marital intimacy is more prominent when middle-aged and elderly stroke patients and spouses are coping with the disease together. This study conducted from December 2020 to July 2021,included 203 stroke patients and their spousal caregivers. They completed the General Data Questionnaire, Dyadic Coping Inventory, the Quality of Relationship Index. Data was analyzed using SPSS 26.0 and Amos 22.0, including descriptive statistics, paired t test, Pearson correlation analysis. And this research used actor-partner interdependence mediation model to explore the relationship of intimacy, among stroke patients and their spousal caregivers.The actor-partner interdependence model revealed actor effects of stress communication, supportive coping, delegated coping.and common coping on intimacy for both patients and their spouses(β =0.572; β = 0.500; β=1.283; β = 0.665, p < 0.05). There were no similar partner effects (p > 0.05). Although patients’ stress communication had a partner effect on spouses intimacy (β = 0.221, p < 0.05), spouses had no similar partner intimacy effect (p >0.05). There were actor and partner effects of negative dyadic coping on intimacy for both patients and their spouses(β = -0.587; β = -0.206, p < 0.05).Our results describe the middle-aged and elderly patients and their spouses as an interactional unit in which positive dyadic coping supports a satisfactory relationship. This study highlights the need for couple-based interventions, including strategies combined with individual and dyadic therapy for both partners.

